# “Omental Cake” in a Patient With Asbestosis Leading to the Diagnosis of Malignant Peritoneal Mesothelioma

**DOI:** 10.7759/cureus.15116

**Published:** 2021-05-19

**Authors:** Alejandro García Martínez, Inmaculada Pavón Guerrero, Lidia Campos Gonzaga

**Affiliations:** 1 Digestive Diseases, Hospital Universitario de Jerez de la Frontera, Jerez de la Frontera, ESP

**Keywords:** malignant peritoneal mesothelioma, abdominal pain, “omental cake”., laparoscopy with peritoneal biopsy, asbestosis

## Abstract

We report a case of a 68-year-old man with medical history of pleural asbestosis and diagnosed with malignant peritoneal mesothelioma. This neoplasm is rare, has a poor prognosis, and is associated with asbestosis in many cases. It manifests clinically insidiously and in relation to the intra-abdominal locoregional effect. Radiological findings are variable, although the finding of “omental cake” by CT scan is characteristic but not pathognomonic, as seen in our case. A biopsy is required for the diagnosis, which can be guided by radiology or surgery. Treatment options available are cytoreductive surgery with intraperitoneal hyperthermic chemotherapy or systemic chemotherapy. However, new therapeutic options are emerging, which are still under development and research.

## Introduction

Malignant peritoneal mesothelioma (MPM) is a rare neoplasm that accounts for 10%-15% of all mesothelioma cases and it is associated up to 50% of the time with asbestosis. It usually presents without differences in terms of sex, with a mean age of 64 years and related to the intra-abdominal locoregional effect, insidiously in the form of abdominal pain or distension [1-4].

## Case presentation

**Figure 1:**
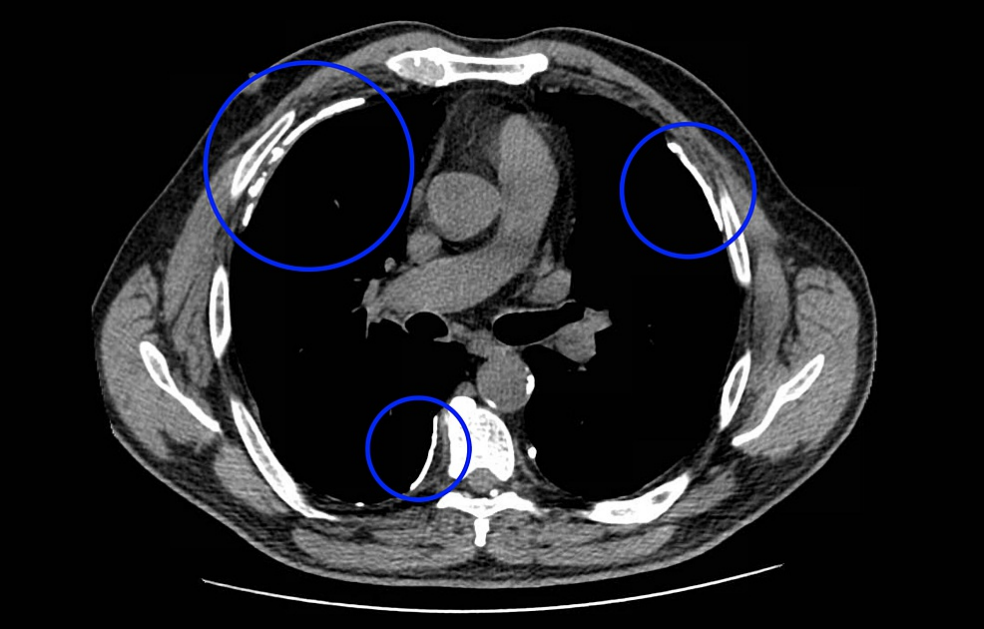
Thorax CT scan: pleural plaques, surrounded by blue circles, as signs of asbestos exposure

We introduce a case of a 68-year-old man, with medical history of being ex-smoker and having pleural asbestosis (Figure 1), who was a shipyard worker exposed to asbestos for 16 years with last exposure about 25 years ago. He sought care in our digestive department due to abdominal pain, especially periumbilical, which was affecting his quality of life, and constipation (fewer than three stools per week and small feces, without pathological products) for six months.

**Figure 2:**
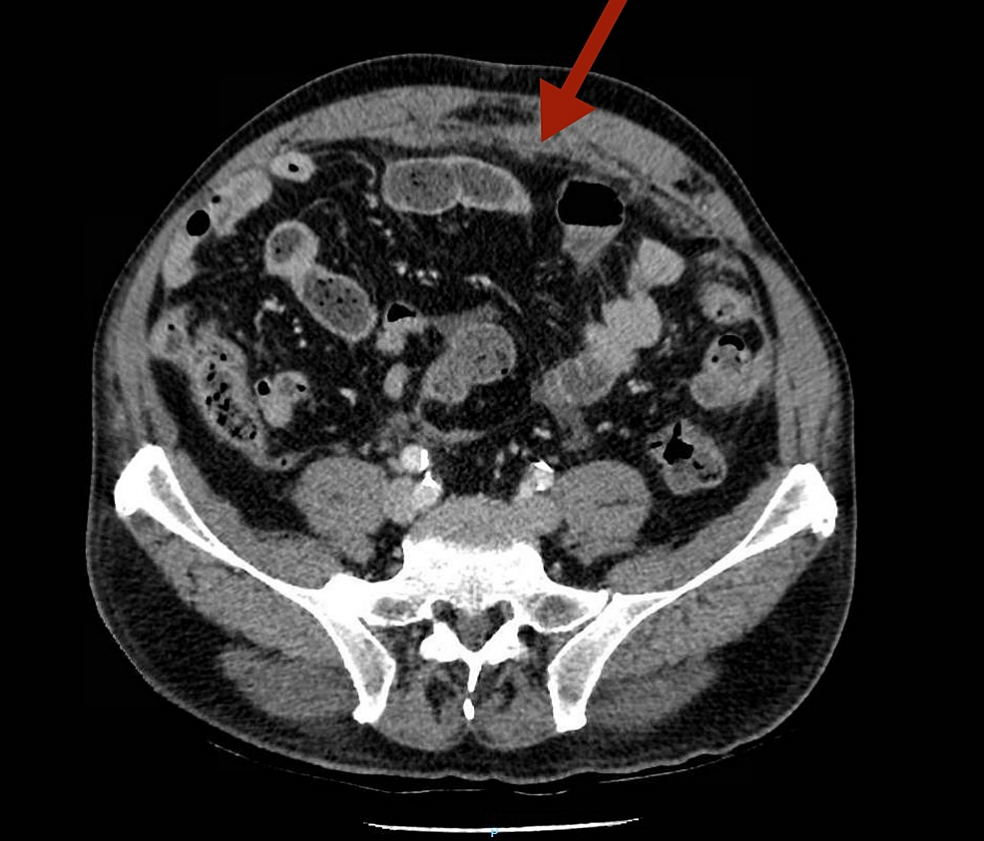
Abdominal CT scan: “omental cake” marked with a red arrow

Laboratory tests (blood count, hemostasis, and biochemistry including creatinine, liver function, lactate dehydrogenase, iron metabolism, cancer antigen [CA] 19.9, and carcinoembryonic antigen) were normal, except for elevation in CA 125 level (85 U/ml). Subsequently, a complete oral digestive endoscopy and colonoscopy were performed at the same time, with the only finding of a colon with poor distensibility. After that, an abdominal CT scan was done, highlighting a thickening of the nodular anterior parietal peritoneum congruent with “omental cake” (Figure 2). Oral digestive endoscopy and colonoscopy were repeated, without other findings that were not described before. So, we decided to take a biopsy of the parietal peritoneum. Initially, biopsy was taken percutaneously by radiological control, which was inconclusive of malignant disease. Therefore, biopsies were obtained by laparoscopy, conclusive of epithelioid malignant mesothelioma and dismissing peritoneal tuberculosis (negative acid-fast bacilli smear, mycobacterial culture, and polymerase chain reaction). After evaluation by a multidisciplinary committee and given the characteristics of our patient, he was referred to oncology department to initiate chemotherapy (cisplatin and pemetrexed). At one-year follow-up, he is still in good clinical condition.

## Discussion

MPM is an entity to consider in the differential diagnosis of abdominal pain, especially if there is a medical record of asbestosis, as is the most common carcinogen identified for mesothelioma. The correlation of mesothelioma to environmental asbestos was first elucidated in the mid-1960s when epidemics in asbestos miners and shipyard workers were exposed (as our patient). However, cases of MPM have been linked less frequently to asbestos exposure compared to the pleural form, as only 33%-50% of patients diagnosed with MPM report any known prior exposure to asbestos compared to greater than 80% in pleural mesothelioma. The latency period between exposure and the development of mesothelioma is approximately 20 years for MPM and 30-40 years for its pleural counterpart. In addition, MPM induced by asbestos generally requires a higher cumulative dose than its pleural counterpart. Other mineral fibers likely play a causative role, with the silicate fiber erionite also being a potent inducer of MPM. In addition, other factors described include therapeutic radiation, papovirus, simian virus, chronic inflammation, and inherited susceptibility [1-3].

Although there is a lack of specificity for MPM in imaging tests, they appear as intraperitoneal masses or diffuse irregular or nodular peritoneal thickening (“omental cake”) on abdominal CT scans, which are characteristic but not pathognomonic. Other findings of MPM are the presence of ascites and very infrequently lymphadenopathy or distant organ involvement. Among tumor markers, CA-125 is elevated in 94% of patients with malignant mesothelioma. However, definitive diagnosis is by biopsy, which can be guided by radiology (CT) or surgery. MPM includes three histologic subtypes: epithelioid (as seen in our case), sarcomatous, and biphasic [1-7]. There is no uniformly accepted classification system for MPM. Due to the infrequency of nodal and metastatic spread, MPM does not fit well into a typical TNM staging paradigm. A novel “TNM” staging system was proposed in 2011 by Yan and associates, based on the extent of peritoneal disease burden (T), intra-abdominal nodal metastasis (N), and extra-abdominal metastasis. The T stage is determined by calculating the peritoneal carcinomatosis index. There are stages I, II, and III, with the corresponding five-year survivals of 87%, 53%, and 29%, respectively. This novel staging system allows better prognostic stratification [2,8].

Other pathologies such as peritoneal carcinomatosis secondary to gastrointestinal or ovarian neoplasm, as well as peritoneal tuberculosis, must be dismissed [1-3].

The selection of the best therapeutic management should be done by a multidisciplinary team specialized in this type of neoplasm, with the current therapeutic options being both cytoreductive surgery with intraperitoneal hyperthermic chemotherapy or systemic chemotherapy [1-4,9]. The patients who benefit from this first, more aggressive therapy are those with good performance status (defined as Eastern Cooperative Oncology Group score 0 or 1), disease distribution favorable for complete or near-complete cytoreductive surgery (extension and depth of the tumor disease does not affect beyond the mesothelial surface), epithelioid histology, female sex (female patients survive longer than male patients), age <60 years, and absence of pretreatment thrombocytosis [1-4, 9-11]. However, for those patients who do not meet these characteristics or whose condition is not operable, systemic chemotherapy (cisplatin and pemetrexed) must be considered, although the disease is usually refractory to the usual regimens [1-4,9]. Another option for first-line treatment, for patients who can tolerate it, is the addition of bevacizumab, a humanized anti-vascular endothelial growth factor, to pemetrexed plus a platinum agent, as it increased overall survival in patients with MPM [1,4]. It is hoped that the molecular characterization of MPM tumors with novel sequencing technologies will lead to the identification of novel molecular targets in this disease. However, nintedanib, an angiokinase inhibitor, has shown in a recent phase II trial to improve the progression-free survival for patients with MPM when administered in combination with pemetrexed and cisplatin. The role of immunotherapy in peritoneal mesothelioma is evolving, but right now there are no available data to recommend using a checkpoint inhibitor alone or in combination as a treatment option for MPM [4,9,12,13].

## Conclusions

MPM is a rare neoplasm but one that we should suspect in a patient with abdominal pain and a medical record of environmental asbestos exposure, as seen in our patient. Findings in the imaging tests are varied but not specific, although they could lead to diagnosis of MPM, which is made by biopsy. Treatment options include cytoreductive surgery with intraperitoneal hyperthermic chemotherapy in selected patients and systemic chemotherapy. However, molecular treatment and immunotherapy are being researched, which could change the management of this cancer. At one-year follow-up, our patient is in good clinical condition and is receiving systemic chemotherapy (cisplatin and pemetrexed), as he could not benefit from cytoreductive surgery with intraperitoneal hyperthermic chemotherapy.
